# Bio-Adrenomedullin and Dipeptidyl Peptidase 3 as Novel Sepsis Biomarkers in the Emergency Department and the Intensive Care Unit: A Narrative Review

**DOI:** 10.3390/medicina61061059

**Published:** 2025-06-09

**Authors:** Ioannis Ventoulis, Christos Verras, Dionysis Matsiras, Vasiliki Bistola, Sofia Bezati, John Parissis, Effie Polyzogopoulou

**Affiliations:** 1 Department of Occupational Therapy, University of Western Macedonia, 50200 Ptolemaida, Greece; iventoulis@uowm.gr; 2 Department of Emergency Medicine, Attikon University Hospital, National and Kapodistrian University of Athens, 12462 Athens, Greece; christos.verras@gmail.com (C.V.); mats.dionysis@gmail.com (D.M.); vasobistola@yahoo.com (V.B.); sofiabezati@gmail.com (S.B.); iparisis@med.uoa.gr (J.P.)

**Keywords:** adrenomedullin, dipeptidyl peptidase 3, biomarkers, sepsis, bio-ADM, DPP3, risk stratification, prognostication

## Abstract

Early recognition and timely treatment of sepsis and septic shock is vital. Despite appropriate management, mortality and morbidity rates remain high. In recent years, many of the research efforts have been directed towards finding novel biomarkers that would rapidly identify, classify and risk-stratify the severity of sepsis in order to achieve prompt and targeted treatment of patients with sepsis and septic shock. Among these biomarkers, adrenomedullin (ADM) in the form of the biologically active fragment (bio-ADM) and dipeptidyl peptidase 3 (DPP3) have recently been in the spotlight. The aim of this narrative review is to summarize current evidence on these two novel biomarkers regarding their clinical utility in diagnosis, prognosis, treatment monitoring and therapy guidance of sepsis and septic shock in the emergency department (ED) and in the intensive care unit (ICU) setting. Bio-ADM seems to be a promising biomarker with respect to the overall management of sepsis (diagnosis, severity prediction, prognosis and treatment monitoring and guidance). On the other hand, DPP3 appears to be useful mainly for sepsis prognosis and for predicting sepsis-induced acute kidney injury. Given their potential clinical utility in sepsis management, the use of these two novel biomarkers, in conjunction with established biomarkers and clinical scores, could lead to the application of refined integrated protocols in the ED and the ICU, which could promptly and effectively inform clinical decision-making in patients presenting with sepsis or septic shock.

## 1. Introduction

Sepsis remains a major healthcare issue worldwide with high morbidity and mortality rates [1]. It constitutes a systemic inflammatory disorder wherein a dysregulated or aberrant host response to infection results in life-threatening organ dysfunction. The most severe form of sepsis is septic shock, which is characterized by profound underlying circulatory, cellular and metabolic abnormalities, which manifest as hypotension requiring vasoactive agents, organ hypoperfusion and increased lactate levels despite adequate fluid resuscitation, all of which pose an even greater cumulative risk of mortality [2]. Additionally, the long-term sequelae of sepsis should not be overlooked. A considerably high proportion of sepsis survivors suffer from a long-term chronic critical illness, which is governed by a protracted course of low-grade inflammation, immunosuppression, persistent organ injury, prolonged protein catabolism, lean tissue wasting and cachexia. This chronic critical phase following the recovery from the initial sepsis event is considered a vulnerable and high-risk state, in terms of adverse events and mortality, and is often marked by long-term cognitive and functional deficits [1,3].

Despite current advances in deciphering molecular mechanisms of sepsis, the management of patients with sepsis has remained rather unchanged over the years. This highlights the fact that there is an unmet need for new personalized and patient-tailored diagnostic and therapeutic strategies [1,4,5]. Taking into account that the management of sepsis is complex, there is an increasing interest in identifying biomarkers that would reflect host response and aid in diagnosis, early recognition of organ dysfunction, risk stratification, prognostication and patient management. An ideal biomarker would further promote antibiotic stewardship, identify suitable patients for a certain therapeutic intervention, risk-stratify them, determine treatment titration and duration, monitor the effectiveness of therapeutic decisions and adjust treatment to the patient’s needs. It could also function as a complementary evaluation tool in conjunction with established score systems of sepsis severity, in order to improve their sensitivity and specificity, mainly in the early stages of disease progression [6,7,8]. Despite research efforts, a biomarker that would serve as a gold standard for the diagnosis and prognosis of sepsis and septic shock has not yet been found [9]. A recent review paper recorded more than 250 biomarkers that have been studied for their potential diagnostic or prognostic role in sepsis. Of note, 80 novel biomarkers have been identified during the period 2010–2020 [7].

The aim of this narrative review is to provide insight into the role of two novel biomarkers, namely adrenomedullin (ADM) and dipeptidyl peptidase 3 (DPP3), in sepsis. An analysis of the underlying pathophysiology of each of these biomarkers will be presented, followed by a summary of existing evidence regarding their clinical utility in the early diagnosis, risk stratification, treatment guidance and prognosis of patients with sepsis and septic shock admitted to the emergency department (ED) or the intensive care unit (ICU). For the purpose of this narrative review, we searched for relevant peer-reviewed journal articles, written in English, via PubMed (MEDLINE) and Scopus databases using a combination of keywords, such as “bio-adrenomedullin”, “bio-ADM”, “adrenomedullin”, “ADM”, “dipeptidyl peptidase 3”, “DPP3”, “sepsis”, “septic shock”, “biomarkers”. The cited bibliography of the retrieved articles was also searched to identify further relevant sources. In vitro and animal studies were excluded from the search, since our goal was to provide an overview of the clinical utility of bio-ADM and DPP3 in patients with sepsis or septic shock.

## 2. Adrenomedullin

### 2.1. Molecular Biology and Pathophysiology

Adrenomedullin (ADM) is a regulatory peptide discovered in 1993 by a group of researchers from Japan. It was first isolated from an extract of human pheochromocytoma, a tumor stemming from the adrenal medulla, based on which it was named [10,11]. It is a freely circulating 52-amino-acid-long peptide encoded by the ADM gene, which is located on human chromosome 11 and contains four exons and three introns [12]. ADM is initially synthesized as a pre-pro-hormone (pre-pro-ADM) containing 185 amino acids. Thereafter, pre-pro-ADM undergoes a multistep cleavage process to eventually yield the final mature product [5,11]. First, cleavage of a 21-residue N-terminal signaling peptide from pre-pro-ADM results in a 164-amino-acid-long peptide called pro-ADM (which corresponds to amino acids 22–185 in the precursor sequence). The next step in the posttranslational process is the cleavage of pro-ADM into the following fragments: pro-ADM N-terminal 20 peptide (PAMP), consisting of amino acids 22–41; midregional pro-ADM (MR-proADM), consisting of amino acids 45–92; adrenotensin, consisting of amino acids 153–185; and a glycine-extended 53-amino-acid-long peptide, consisting of amino acids 95–147. The latter fragment, namely the glycine-extended 53-amino-acid-long peptide, is converted to the mature and biologically active form of ADM (bio-ADM) via enzymatic amidation of the glycine extension located at its C-terminus [5,10,11,13,14].

ADM circulates in the plasma in two molecular forms: the glycine-extended form with 53 amino acids, which is the prevailing form within the plasma pool, and the mature active form (bio-ADM) with 52 amino acids, which is the end-product generated after glycine amidation [10,11]. Bio-ADM has a very short half-life (approximately 22 min) [5,15], owing to its fast degradation by plasma proteases [5], as well as to the formation of complexes in the blood with complement factor H, also known as “ADM binding protein 1 (AMBP-1)” [10]. On the contrary, MR-proADM, the apparently inactive 48-amino-acid-long fragment of cleaved proADM, has a half-life of several hours, which renders it a more stable molecule. Since MR-proADM occurs in a stoichiometric ratio of 1:1 with ADM, it reliably reflects the levels and activity of ADM and can thus be used as a surrogate for ADM [16,17].

Although the highest concentrations of ADM are found in the adrenal medulla, cardiac atria and lungs, both ADM and its receptors are in fact expressed in almost all human tissues [5]. ADM is widely distributed in various body fluids, organs and epithelial surfaces [10,11]. Endothelial cells and vascular smooth muscle cells (VSMCs) are among the various cell types that produce and secrete ADM. Its production is thought to be constant, given that it is not stored in secretory granules, but is rather secreted immediately upon formation [5,11]. ADM is metabolized through two distinct degradation pathways. The first one involves the enzymatic cleavage of its N-terminus by several circulating and membrane-bound proteases, with neprilysin being the predominant endopeptidase responsible for its degradation. The second one entails the internalization and lysosomal degradation of activated ADM receptor complexes, which takes place mainly in the lungs, since the pulmonary circulation is one of the principal clearance sites for ADM [5,17,18].

ADM exerts its biological effects upon binding to its receptors, namely ADM1 and ADM2. These receptors are heterodimeric complexes comprising two different peptides: a calcitonin receptor-like receptor (CRLR), which is a polypeptide with seven transmembrane-spanning domains; and a specific receptor activity-modifying protein (RAMP), which is a protein with a single transmembrane domain. RAMP can be either RAMP2 or RAMP3. When CRLR is combined with RAMP2, it gives rise to the ADM1 receptor, whereas the ADM2 receptor is formed when CRLR molecularly associates with RAMP3 [5,11,19]. The biological effects of ADM are pleiotropic and include immune modulation, anti-inflammation, regulation of metabolism and growth, hormone regulation, adjustment of fluid and electrolyte balance, neurotransmission, bronchodilation, as well as several cardiovascular effects, with potent vasodilation and stabilization of the endothelial barrier being the most remarkable ones [5,10,11,19]. With regard to its vascular actions, ADM regulates vascular tone by binding to its receptors which are located on both endothelial cells and VSMCs, thereby netting a vasodilatory effect through two distinct pathways. The first one involves indirect relaxation through endothelial cells by inducing endothelial nitric oxide synthase (eNOS) activity and increasing local nitric oxide (NO) release, whereas the second one involves direct relaxation of VSMCs by increasing cyclic adenosine monophosphate (cAMP) or activating potassium channels [5,19]. Additionally, ADM plays a major role in the development, stability and integrity of the endothelial barrier, which is crucial for regulating vascular permeability, electrolyte balance and homeostasis, local vascular reactivity, as well as local and systemic inflammatory signaling [5,10,11].

ADM has been shown to be implicated in the host defense mechanisms during septic response [5,10,11,20,21]. Sepsis and septic shock are characterized by circulatory, cellular and metabolic derangements. Prominent pathophysiological features include vasodilation and loss of vascular integrity characterized by vascular leakage with extravasation of intravascular fluids into the interstitium and the formation of interstitial edema. The ensuing endothelial dysfunction culminates in persisting vascular hyperpermeability, diminished microvascular reactivity and overt myocardial dysfunction, which mark the transition from the hyperdynamic to the hypodynamic phase of septic shock and eventually result in hypotension, multiple organ dysfunction and finally death [22,23,24,25]. Throughout the course of these pathophysiological processes, high ADM concentrations are observed in the circulation. In fact, the elevation in ADM levels is proportional to the disease severity, the degree of organ dysfunction and the mortality risk [5,12,20,26]. Elevated ADM levels are ascribed to a dual mechanism, which includes an increase in ADM production by numerous organs, on one hand, and a decrease in its clearance, on the other. Enhanced ADM synthesis is induced by several factors, including lipopolysaccharide (LPS), tumor necrosis factor (TNF) and other proinflammatory cytokines, thrombin, thyroid hormones and glucocorticoids [11].

It is noteworthy that ADM has been characterized as a “double-edged sword” in sepsis [27], given that elevated ADM levels tend to restore the function of the endothelial barrier and are thus considered a beneficial part of the host defense mechanism, but at the same time, high ADM concentrations exert deleterious effects by inducing vasodilation and hypotension [26]. To this end, the biological role of ADM remains controversial in sepsis, since it has been paradoxically observed that the administration of ADM may exert favorable effects, while at the same time, the administration of an antibody against ADM may also be beneficial [27,28]. Indeed, experimental studies in septic animals have demonstrated that exogenous administration of ADM reduces endothelial hyperpermeability, mitigates end-organ damage and improves survival [29,30,31,32]. Moreover, in an animal model of sepsis, it has been reported that the administration of ADM together with its serum binding protein AMBP-1 hampered the transition to more severe phases of sepsis, prevented progression to septic shock and improved sepsis-induced mortality by exerting beneficial effects on cardiovascular response during sepsis and thus attenuating tissue injury [21]. On the other hand, the administration of anti-ADM antibodies has also shown favorable outcomes in preclinical studies of sepsis [33,34,35]. Interestingly, in a murine model of sepsis, Struck et al. studied the effects of various anti-ADM antibodies directed at different epitopes of ADM and concluded that the best survival benefit was conferred by a certain type of anti-ADM antibody which targeted the N-terminal region of the ADM peptide, but at the same time exhibited weak antagonist activity with only partial inhibition of ADM signaling [36]. Subsequently, research has focused on the effects of the non-neutralizing antibodies which are directed at the N-terminal region of ADM and do not completely inhibit ADM action. These non-neutralizing antibodies have shown some promising preliminary results [34,35,37]. It is believed that they act by binding to ADM and preventing its diffusion into the interstitial space, thus confining it in the intravascular compartment and increasing its bioactive levels in the circulation. Concomitantly, antibody binding limits the hydrolysis of ADM, and therefore, increases its half-life. By achieving higher intravascular levels of ADM, its beneficial effects on the endothelial barrier are amplified, whereas its vasodilatory action on VSMCs in the interstitial space is diminished. Consequently, this translates into a reduction of vasopressor requirements, attenuation of end-organ injury and improved survival [5,38,39,40]. Regardless of the underlying mechanisms, several clinical studies have shown that ADM levels have diagnostic and prognostic value in sepsis.

### 2.2. Bio-ADM as a Diagnostic Tool for Sepsis

The diagnostic performance of bio-ADM, that is the ability to discriminate septic from non-septic patients, was a primary endpoint in a prospective observational trial comprising 98 patients admitted to the ICU, 56 of whom had sepsis. Bio-ADM levels were measured at two different time points: upon admission and on day 3 post-admission. Upon admission, bio-ADM levels were significantly higher in septic compared to non-septic patients. However, on day 3 post-admission, the levels were no longer statistically different between septic and non-septic patients, thus suggesting that bio-ADM is capable of identifying sepsis within a limited time frame from sepsis onset, restricted at the very early stage of sepsis. The diagnostic cut-off value of bio-ADM was determined at 31.2 pg/mL. Regarding its diagnostic accuracy, it was observed that bio-ADM performed better than other markers of sepsis during receiver operating characteristic (ROC) analysis. Indeed, the area under the curve (AUC) of bio-ADM for diagnosing sepsis was 0.848, which was higher than the respective AUCs of procalcitonin (0.828), presepsin (0.682), lactate (0.66) and sequential organ failure assessment (SOFA) score (0.772). The corresponding sensitivity and specificity for sepsis diagnosis were 88.1% and 67.9% [41].

The diagnostic value of bio-ADM has also been explored as a secondary outcome in a retrospective multicenter observational study from Sweden, which included 1867 patients admitted to the ICU, of whom 632 had sepsis and 267 had septic shock. Median bio-ADM levels were 40 pg/mL in the entire ICU cohort, 74 pg/mL in patients with sepsis, 107 pg/mL in patients with septic shock, and 29 pg/mL in non-septic patients. In the entire ICU cohort, after adjusting for disease severity, increased bio-ADM levels were associated with an increased risk of having sepsis with an odds ratio (OR) of 1.78 [95% confidence interval (CI) 1.64–1.94]. The ability of bio-ADM to diagnose sepsis among ICU patients was modest, yielding an AUC of 0.76 (0.73–0.78). Likewise, a Youden’s index-derived threshold of 37 pg/mL yielded a sensitivity of 61% and a specificity of 80% for identifying sepsis, with a positive predictive value (PPV) of 51% and a negative predictive value (NPV) of 86% [42].

The main parameters for bio-ADM-based diagnosis of sepsis, derived from these two studies, are presented in Table 1.

### 2.3. Bio-ADM as a Risk Stratification Tool and a Marker of Morbidity

The role of bio-ADM as a risk stratification tool and a marker of morbidity was examined in a well-defined subset of ICU patients with severe sepsis or septic shock, derived from the Albumin Italian Outcome Sepsis (ALBIOS) trial. ALBIOS was a multicenter randomized controlled trial that enrolled 1818 septic patients from 100 Italian ICUs, 956 of whom participated in a predefined biomarker substudy with measurement of bio-ADM levels on days 1, 2 and 7 after ICU admission. In this subset of patients, it was shown that high levels of bio-ADM could predict incident cardiovascular dysfunction, shock and multiple organ failure. Bio-ADM levels on day 1 could also predict the intensity of hemodynamic support therapy, in terms of requirements in vasopressors, inotropes and fluids, over the first week of treatment. Likewise, the time course of serum lactate levels could be predicted by baseline bio-ADM levels, since patients in the upper tertile of bio-ADM (≥154 pg/mL) exhibited significantly higher lactate concentrations over the first 7 days of treatment. Patients presenting with septic shock demonstrated significantly higher baseline bio-ADM levels than those presenting with sepsis. Moreover, higher baseline concentrations of bio-ADM were correlated with a higher likelihood of developing incident shock, as well as with a more protracted length of hospital stay and a higher rate of renal replacement therapy. Higher bio-ADM levels were also strongly associated with higher SOFA score, ICU admission after emergency surgery, positive blood culture, higher heart rate, higher central venous pressure (CVP), lower mean arterial pressure (MAP) and higher serum creatinine [43].

Similar findings were reported in the Adrenomedullin and Outcome in Sepsis and Septic Shock 1 (AdrenOSS-1) trial, which was a prospective observational study conducted in 24 centers from five European countries (France, Belgium, the Netherlands, Italy and Germany). The study included 583 patients admitted to the ICU with sepsis or septic shock and examined the relationship of bio-ADM levels with the following secondary outcomes: organ failure as assessed by the SOFA score, need for organ support, need for vasopressors and inotropes, fluid balance and need for renal replacement therapy. Patients with septic shock had significantly higher bio-ADM levels (114.4 pg/mL) compared to patients with sepsis (57.5 pg/mL). Bio-ADM levels upon ICU admission were found to be correlated with the baseline SOFA score. Furthermore, baseline bio-ADM levels were strongly associated with the need for organ support, as well as with the duration of organ support. With regard to circulatory support, there was an almost linear relationship between bio-ADM levels and cardiovascular SOFA subscore. A similar relationship was evident between bio-ADM levels and duration of vasoactive therapy. Patients presenting with high bio-ADM levels were more likely to require vasopressors both upon admission and over the following 7 days. Even more so, patients with high bio-ADM levels needed greater doses of vasopressors compared to patients with low bio-ADM. Moreover, higher bio-ADM levels were associated with more aggressive volume resuscitation (>5 L) over the first 48 h. Additionally, patients with higher bio-ADM concentrations were more likely to require renal replacement therapy or have a prolonged ICU stay [26].

Along the same lines, in the study by Lundberg et al., elevated bio-ADM levels upon ICU admission were shown to be correlated with a higher need for organ support. As a matter of fact, higher bio-ADM levels were found to be associated with an increased need for continuous renal replacement therapy (OR 1.97 [1.64–2.36]), as well as with an increased need for circulatory support (OR 1.33 [1.17–1.50]) in patients with sepsis [42].

Two years later, the same group of investigators conducted a single-center prospective observational study in the ED setting. They studied 594 patients presenting to the ED with sepsis and investigated the association of bio-ADM with multiple organ failure and ICU admission as secondary study outcomes. They reported that bio-ADM levels were correlated with the severity of organ failure, as indicated by the number of failing organ systems. Median bio-ADM concentrations were significantly lower in patients without organ failure (31 [21–44] pg/mL) compared to patients with intermediate organ failure defined as failure of 1–3 organ systems (45 [31–72] pg/mL), whereas the highest bio-ADM levels were observed in patients with severe organ failure defined as 4 or more failing organ systems (81 [56–156] pg/mL). In both uni- and multi-variate regression analysis, increasing bio-ADM levels were significantly associated with the development of severe multiple organ failure. In a similar fashion, patients admitted to the ICU had significantly higher bio-ADM levels (77 [42–133] pg/mL) than those not requiring ICU admission (41 [28–61] pg/mL). With increasing bio-ADM levels, patients had significantly higher odds of being admitted to the ICU, even after adjusting for several clinical variables including age and site of infection. On the other hand, patients with low levels of bio-ADM were more likely to have less severe disease and be discharged from the ED. The authors concluded that bio-ADM could be potentially used in the ED clinical setting for early risk stratification of unselected patients with sepsis [20].

In another study by Casalboni et al., which included 177 patients with sepsis and was conducted exclusively in the ED setting, it was reported that bio-ADM levels were significantly correlated with lower MAP, elevated lactate levels, higher SOFA score, higher serum creatinine, reduced urinary output, lower arterial pH and higher procalcitonin concentrations. In other words, bio-ADM was found to be correlated with parameters of hemodynamic and renal compromise, thereby indicating multi-organ failure and disease severity requiring ICU admission [44]. Further evidence on the potential role of bio-ADM as a marker of morbidity was provided by a recent prospective multicenter study, which sought to examine the performance of various biomarkers in terms of improving sepsis diagnosis and identifying incident organ dysfunction in an unselected cohort of 1426 patients presenting to the ED with a quick SOFA (qSOFA) score ≥1. It was demonstrated that bio-ADM was associated with disease severity and organ dysfunction, as reflected by the need for ICU admission and the requirement for mechanical ventilation, respectively. A correlation was also found between bio-ADM and length of hospital or ICU stay [45].

Likewise, in a prospective observational study of 101 patients presenting to the ED with sepsis, bio-ADM levels upon ED presentation were found to be strongly associated with disease severity, as evidenced by a positive correlation between baseline bio-ADM levels and Acute Physiology and Chronic Health Evaluation II (APACHE II) score. Baseline bio-ADM levels were significantly higher in patients with severe sepsis or septic shock (93 [50–232] pg/mL) than in patients with sepsis (48 [32–72] pg/mL). It was also observed that baseline bio-ADM levels were negatively correlated with MAP. More importantly, bio-ADM could discriminate patients requiring hemodynamic support from those who did not require vasopressors, with median bio-ADM levels being 129 [83–264] pg/mL in the former group vs. 48 [32–75] pg/mL in the latter [46].

Besides, in the study by Kim et al., bio-ADM was found to predict sepsis severity and organ failure in a mixed cohort of 215 septic patients from both the ED and the ICU. Bio-ADM levels were significantly higher in patients with septic shock (110.3 pg/mL) compared to patients with sepsis (45.3 pg/mL). Likewise, patients requiring vasopressors had significantly higher bio-ADM concentrations (99.3 pg/mL) than those without need of vasopressors (44 pg/mL). Moreover, with increasing bio-ADM levels, a stepwise increase in the number of failing organ systems was observed. Bio-ADM levels were significantly associated with the failure of specific organ systems in the SOFA scoring, especially with cardiovascular, renal, coagulation and liver subscores [12].

### 2.4. Bio-ADM as a Tool for Guiding Treatment

In the ALBIOS study, Caironi et al. found that baseline bio-ADM levels were associated with the intensity of the hemodynamic support therapy during the clinical course of sepsis, since higher baseline bio-ADM concentrations could predict more positive fluid balance and higher requirements in vasoactive drugs. Based on these observations, the authors speculated that bio-ADM levels and their time-course trajectory could serve as a potential target during sepsis resuscitation and guide hemodynamic treatment by adjusting fluid administration and doses of vasopressors, in order to avoid excessive fluid overloading while achieving adequate hemodynamic support at the same time. In this regard, sequential measurement of bio-ADM could be proposed as a tool to clinically monitor the efficacy of the therapeutic measures applied during the time-course of sepsis, as well as a tool to guide treatment by individualizing hemodynamic support therapy [43].

In the AdrenOSS-1 trial, it was shown that serial measurements of bio-ADM levels upon ICU admission and after 48 h provided prognostic information about subsequent organ recovery. As a matter of fact, a decrease in bio-ADM levels towards normal values (<70 pg/mL) over the first 2 days was associated with the reversibility of organ failure and recovery of organ function within the first week of ICU hospitalization. More importantly, the decrease in bio-ADM concentrations preceded the improvement in the total SOFA score, thus serving as a better and earlier prognostic marker of organ recovery. An early drop in circulating bio-ADM levels by day 2 was mainly associated with subsequent restoration of cardiovascular function and reduced need for vasopressor use by day 7, whereas persistently elevated bio-ADM levels on day 2 were correlated with prolonged organ dysfunction. Therefore, it could be assumed that early changes in bio-ADM levels during serial measurements could inform clinicians about the efficacy of ongoing sepsis treatment and further guide decision-making about hemodynamic support, fluid balance and potential changes required in the therapeutic plan [26].

### 2.5. Bio-ADM as a Prognosticator of Mortality

The prognostic utility of bio-ADM in terms of predicting short- and/or long-term mortality risk has been an endpoint in several studies. Caironi et al. demonstrated that baseline bio-ADM levels were independently associated with 90-day mortality in a cohort of 956 ICU patients with sepsis or septic shock. They observed that progressive elevations in baseline bio-ADM concentrations were accompanied by a stepwise incremental increase in mortality. In fact, they reported a 1.9-fold increase in mortality rate from the lower to the upper tertile of baseline bio-ADM concentrations. Furthermore, the authors showed that the time-course of bio-ADM levels over the first 7 days was a strong and independent predictor of subsequent mortality at 90 days. When bio-ADM levels remained high or increased over time, the prognosis was grim with a high 90-day mortality rate, whereas when bio-ADM levels decreased to a value lower than 110 pg/mL, the outcome was profoundly better with a marked reduction in 90-day mortality [43].

These results were corroborated by the findings of Mebazza et al. who investigated the relationship between bio-ADM and 28-day mortality in the AdrenOSS-1 trial and confirmed the strong association between baseline bio-ADM levels and short-term mortality, as well as the prognostic value of repeated bio-ADM measurements during ICU hospitalization. By using a Cox proportional hazards regression model adjusted for various parameters (age, gender, comorbidities, lactate, diagnosis of sepsis or septic shock), the authors showed that bio-ADM levels upon ICU admission were independently and strongly associated with 28-day mortality with an adjusted standardized hazard ratio (HR) of 1.6 [1.1–2.5] (*p* = 0.0004). Moreover, it was demonstrated that baseline bio-ADM levels conferred added prognostic value on top of established ICU severity scores, such as the APACHE II or the SOFA scoring system. Furthermore, it was also shown that changes in bio-ADM concentrations during the initial 48 h exhibited predictive value for 28-day mortality. When there was a decrease in bio-ADM levels to normal values (<70 pg/mL) on day 2, the 28-day mortality was low, whereas mortality was high when bio-ADM levels increased or remained elevated. Even patients with initially high bio-ADM levels on admission had a favorable 28-day outcome once their bio-ADM levels had decreased to normal values within 48 h. In fact, their mortality rate was similar and, interestingly enough, slightly lower than the mortality of patients whose bio-ADM levels remained constantly low (9.5% vs. 10.5%, respectively) [26].

Lundberg et al. conducted a retrospective study in a cohort of 1867 patients hospitalized in the ICU. The primary endpoint was to examine whether there was an association between bio-ADM levels upon ICU admission and 30-day mortality in both patients with sepsis and the overall ICU cohort. Within the sepsis cohort, it was observed that bio-ADM levels were significantly higher in non-survivors compared to survivors. During regression analysis, elevated bio-ADM levels were associated with increased mortality with an OR of 1.23 [1.07–1.41] for patients with sepsis and an OR of 1.22 [1.12–1.32] for the entire ICU cohort. It was also found that a two-fold increase in bio-ADM levels resulted in a 22–23% increased OR for death. In patients with sepsis, the association with mortality remained significant even after adjusting for lactate levels, indicating that bio-ADM levels convey additional prognostic information in sepsis. In the sepsis cohort, a bio-ADM cut-off of 70 pg/mL was able to discriminate survivors from non-survivors with 60% sensitivity and 50% specificity. However, the application of a Youden’s index-derived cut-off of 108 pg/mL in the Kaplan–Meier curve for the sepsis cohort resulted in better specificity (68%), albeit at the expense of lower sensitivity (48%) [42].

Apart from the ICU setting, bio-ADM levels were also found to be associated with 28-day mortality in the ED setting in a cohort of 594 patients with sepsis. Non-survivors exhibited significantly higher bio-ADM levels compared to survivors (63 [42–132] pg/mL vs. 36 [26–56] pg/mL, respectively). The association of bio-ADM with 28-day mortality remained significant even after adjusting for multiple clinical variables, such as age, prior cardiovascular disease, body mass index and site of infection. Regarding the prediction of 28-day mortality, bio-ADM yielded a significantly higher AUC (0.73) compared to lactate (0.57), C-reactive protein (CRP) (0.59) or creatinine (0.62). Yet, the predictive ability of bio-ADM per se was only modest. When bio-ADM was added to a baseline mortality prediction model, which included the aforementioned clinical parameters and biomarkers, it resulted in a significant increase in the AUC for predicting mortality from 0.80 [0.75–0.85] to 0.86 [0.81–0.91] [20].

The prognostic value of bio-ADM in the ED setting was also investigated in a prospective observational study, which included 177 patients presenting to the ED with sepsis. Bio-ADM was measured upon ED presentation by means of a point-of-care assay. Bio-ADM levels were significantly higher in non-survivors (55 pg/mL) compared to survivors (44 pg/mL), *p* < 0.001. Bio-ADM levels were significantly associated with 30-day mortality. When using a bio-ADM cut-off of 65 pg/mL during Kaplan–Meier survival analysis, the HR of dying at 30 days for patients with baseline bio-ADM levels above the cut-off was 2.14 ± 0.36. During ROC analysis, it was shown that bio-ADM exhibited the best sensitivity and specificity for predicting 30-day mortality, yielding an AUC of 0.73, which was superior to the AUC (0.60) of proenkephalin and the AUCs (0.68) of established prognostic scores, such as SOFA and National Early Warning Score (NEWS), while it was equivalent to the AUC (0.73) of Rapid Emergency Medicine Score (REMS). When comparing the ROC curve of bio-ADM with those of commonly used biomarkers, namely CRP and procalcitonin, it was observed that bio-ADM outperformed the other biomarkers in terms of prognostic performance, resulting in an AUC of 0.695 compared to an AUC of 0.511 for procalcitonin and an AUC of 0.479 for CRP [44]. Besides, bio-ADM was shown to provide prognostic information on 28-day all-cause mortality in a cohort of 1426 patients presenting to the ED with suspected organ dysfunction based on a qSOFA score ≥1, 29.2% of whom were eventually diagnosed with sepsis [45].

Furthermore, bio-ADM levels were shown to be associated with short-term mortality in a cohort of 101 patients admitted to the ED with sepsis. Patients who died over an observation period of 28 days had significantly higher bio-ADM levels upon admission (84 [48–232] pg/mL) than those who survived (50 [31–77] pg/mL). Of note, baseline bio-ADM concentrations were found to be associated with the cause of in-hospital death, since they were significantly higher in patients who died due to septic shock (177 [77–289] pg/mL) compared to patients who died due to other causes (54 [45–96] pg/mL). Baseline bio-ADM levels could predict 28-day mortality with an HR of 2.6, yielding an AUC of 0.69, whereas the AUC of APACHE II score was 0.75. In a multivariable Cox regression model, it was shown that bio-ADM could predict mortality independently of APACHE II and provided additional prognostic information on top of the APACHE II score. It was also demonstrated that serial measurements of bio-ADM over the first 4 days of hospitalization resulted in improvement in the prognostic performance of bio-ADM, since the C-index for predicting mortality improved from 0.69 (when using only baseline bio-ADM levels) to 0.75 (when superimposing bio-ADM levels on day 4 on top of baseline bio-ADM levels). As a matter of fact, when considering the group of patients with baseline bio-ADM concentrations above 70 pg/mL, their 28-day survival rate was 55%. When bio-ADM levels on day 4 remained above 70 pg/mL, their survival rate was 36%, whereas when bio-ADM levels had decreased below 70 pg/mL by day 4, then the survival rate was 100% [46].

A Korean study included a mixed cohort of 215 septic patients from both the ED (123 patients) and the ICU (92 patients) and explored the role of bio-ADM as a prognosticator of mortality. Bio-ADM levels were significantly higher in non-survivors (137.8 pg/mL) compared to survivors (55.3 pg/mL). When bio-ADM concentrations were divided into quartiles (Q1, Q2, Q3 and Q4), a stepwise increase of 30-day mortality was observed across quartiles. Patients belonging to the first bio-ADM quartile (Q1 < 36.5 pg/mL) exhibited the lowest mortality rate (5.6%), whereas patients in the fourth quartile (Q4 ≥ 139 pg/mL) demonstrated the highest mortality rate (61.1%). Mortality rates for the intermediate quartiles (36.5 pg/mL ≤ Q2 < 75.8 pg/mL and 75.8 pg/mL ≤ Q3 < 139.0 pg/mL) were 11.1% and 45.3%, respectively. Patients with bio-ADM levels in the Q3 and Q4 groups demonstrated significantly higher HRs of dying compared to the Q1 and Q2 groups. Notably, bio-ADM quartile groups were able to stratify the survival probability not only in the overall patient cohort, but also within the subgroups of patients with sepsis only (but not shock) and with septic shock. Accordingly, very high bio-ADM levels in the subgroup of patients with sepsis only (without shock) could identify those with a very high mortality risk, and inversely very low bio-ADM levels in the subgroup of patients with septic shock could distinguish those who ran a low mortality risk. More importantly, neither SOFA cardiovascular subscore nor lactate quartiles could predict and stratify mortality risk within each subgroup of patients with sepsis and septic shock as consistently as bio-ADM quartiles could. Regarding the ability to predict short-term mortality in the overall cohort, bio-ADM demonstrated similar predictive performance to the SOFA score in terms of AUC (0.827 vs. 0.830, respectively). However, during Cox proportional hazards regression, bio-ADM showed a higher relative risk (3.6) compared to SOFA score (1.2), thereby implying a greater prognostic effect of bio-ADM levels on 30-day mortality than SOFA [12].

Interestingly, a study investigating the prognostic role of bio-ADM, in terms of predicting mortality as a secondary endpoint, reported temporal time-dependent variations with regard to the prognostic performance of bio-ADM in ICU patients with sepsis. It was observed that bio-ADM levels could predict 28-day mortality on day 3 post-admission, but not on day 1. Upon admission, bio-ADM levels did not differ significantly between survivors and non-survivors. However, on day 3 post-admission, bio-ADM levels were significantly higher in non-survivors compared to survivors. In a similar fashion, the AUC of bio-ADM for predicting 28-day mortality in patients with sepsis was low upon admission (0.551) and became significant only on day 3 post-admission (0.892), yielding a sensitivity of 83.3% and a specificity of 88.2%. The respective cut-off of bio-ADM for predicting 28-day mortality on day 3 was 32.4 pg/mL. These findings might be explained by the fact that persistently elevated levels of bio-ADM on day 3 probably imply failure of treatment and ongoing organ damage, both of which portend poor prognosis [41].

Table 2 summarizes the main characteristics and findings of the studies that have examined the role of bio-ADM as a biomarker of diagnosis, risk stratification, prediction of severity, morbidity and mortality, and treatment guidance in patients with sepsis.

## 3. Dipeptidyl Peptidase 3 (DPP3)

### 3.1. Molecular Biology and Pathophysiology

Dipeptidyl peptidase 3 (DPP3) belongs to the dipeptidyl peptidase family and has been specifically designated as a member of the M49 family of metallopeptidases [47,48]. It is officially registered as EC3.4.14.4 in the nomenclature and classification list of the International Union of Biochemistry and Molecular Biology [48]. It was first isolated from the bovine anterior pituitary gland in 1967 and it was the third member of the family to be discovered, hence the name DPP3 [48,49]. Since then, it has been isolated from numerous prokaryotic and eukaryotic species, including microorganisms, parasites, yeasts, insects and mammals [48,49]. Several names have been assigned to it, such as dipeptidyl arylamidase III, dipeptidylaminopeptidase III, enkephalinase B or red cell angiotensinase [47,48], while recently it has been shown that an oligopeptidase, which was lately isolated from synaptosomes of bovine and rat brains and was coined neuron bound extracellular metallopeptidase 3 (NEMP3), is actually the same enzymatic protein as DPP3 [50]. DPP3 is widely expressed in almost all tissues, including blood cells (erythrocytes, leucocytes), lung, heart, blood vessels, kidney (renal epithelial cells), adrenal glands, intestines, skeletal muscle, skin, brain, eye, liver, spleen and placenta [5,48,49].

DPP3 is a zinc-dependent metallopeptidase which contains a catalytic zinc-binding domain consisting of a unique HEXXGH conserved catalytic motif [49]. It specifically cleaves dipeptides from the N-terminus of oligopeptides with a length of four to ten amino acids. DPP3 cannot exert any enzymatic action on peptides with more than ten amino acids, whereas peptides with only three amino acids are not ideal substrates since they are poorly hydrolyzed by DPP3 [5,48]. It has been primarily classified as a cytosolic enzyme; yet, it has also been reported to exist in membrane-bound forms. Additionally, translocation of DPP3 into the nucleus has been described under conditions of oxidative stress, whereas DPP3 can also be found extracellularly, specifically in the cerebrospinal fluid, seminal plasma and retroplacental serum [5,48]. The presence of DPP3 has also been demonstrated in human plasma, in which it can be accurately detected and quantified by employing highly specific assays [51]. Through the use of these assays, increased levels of circulating DPP3 in plasma have been observed under inflammatory and shock conditions [5,48,51]. The half-life of DPP3 in plasma is approximately 70 min [5,51].

The enzymatic function of DPP3 involves the cleavage of a dipeptide fragment from the N-terminus of various bioactive peptide substrates, including angiotensins, enkephalins, endorphins, other peptides and dipeptidyl derivatives [48]. The biological enzymatic significance of DPP3 is substantiated by the fact that its catalytic domain has been highly preserved among species during the evolution process [5,47]. Regarding its role, DPP3 is involved in an array of biological events, which include protein turnover and metabolism, bioactive peptide homeostasis, cell cycle regulation, cancer, blood pressure control, inflammation, immunomodulation, cytoprotection against oxidative stress, hormone processing, pain regulation, learning and memory processes, and emotional and behavioural modulation [47].

The primary physiological action of DPP3 is the modulation of the renin-angiotensin-aldosterone system (RAAS), through hydrolysis of its substrates, namely angiotensins [48]. Of note, DPP3 is responsible for the degradation of both angiotensin II (Ang II) and angiotensin (1–7), which represent two distinct antagonistic pathways [52]. On the one hand, Ang II is the main endogenous ligand of the classic RAAS pathway that primarily results in the activation of the Ang II type 1 (AT1) receptors, which mediate vasoconstriction, water and sodium retention, increased release of vasopressin, increased sympathetic output, as well as cardiac and vascular hypertrophy and fibrosis [53,54,55]. On the other hand, angiotensin (1–7) is derived from Ang II through conversion by the angiotensin II-converting enzyme 2 (ACE2) and, upon its production, it activates the Mas receptor, which in turn results in vasodilation, increased renal blood flow, increased natriuresis and reduced myocardial fibrosis [52]. This ACE2-Angiotensin (1–7)-Mas receptor (AAM) axis is the antagonistic pathway of the classic RAAS axis and DPP3 is implicated in both axes, thereby affecting multiple physiological and pathophysiological processes in the heart, kidney and vasculature [48,52,56]. By means of interaction with both angiotensin pathways, DPP3 exerts its effects on the hemodynamics and physiology of the cardiovascular system, mainly by acting as a depressant of the cardiovascular system and by lowering vascular tone and blood pressure, although the exact mechanisms by which cardiovascular function is affected remain unclear [5,48,49].

Besides, the precise mechanisms through which DPP3 is released into the extracellular space remain elusive, given the fact that DPP3 is primarily located intracellularly in the cytosol [57]. Normally, low levels of DPP3 are detected in the plasma of healthy individuals, but under acute stress conditions the concentrations of DPP3 in the blood circulation are dramatically elevated [51]. Indeed, increased plasma DPP3 levels have been reported in critically ill patients with sepsis [51], septic shock [51,58], cardiogenic shock [59] and burn-induced vasodilatory shock [60]. Based on these observations, it has been postulated that, under critical pathological conditions which impair tissue perfusion and cause cellular injury and necrosis, elevated DPP3 levels in the plasma may be attributed to the fact that cytosolic DPP3 is released into the bloodstream as a result of membrane cell disruption, lysis and ongoing cell death [5,48]. In turn, DPP3 in plasma leads to accelerated degradation of circulating Ang II and thus results in further deterioration of tissue perfusion by competing the vasopressor effects of endogenous Ang II, reducing vascular tone and exerting cardiodepressant effects [5,48,56,61].

### 3.2. DPP3 as a Risk Stratification Tool and a Marker of Morbidity

In a prospective observational multinational study from five European countries, DPP3 plasma levels were measured in 581 patients admitted to the ICU due to severe sepsis or septic shock. Median levels of DPP3 upon admission were 26.5 [16.2–40.4] ng/mL. Notably, significantly higher DPP3 baseline levels were observed in patients with septic shock (29.1 [18–48.2] ng/mL) compared to patients with severe sepsis (23.2 [15.2–35.1] ng/mL). High DPP3 levels at baseline (defined as levels above the third quartile >40.4 ng/mL) were associated with higher SOFA and APACHE II scores, longer ICU stay and worse metabolic parameters, including higher lactate levels and lower arterial pH. They were also associated with worse renal function, as evidenced by higher creatinine and urea levels, worse urine output and more positive fluid balance. Furthermore, high DPP3 baseline levels were correlated with increased heart rate and higher N-terminal pro B-type natriuretic peptide (NT-proBNP) levels, suggestive of worse cardiac function. An association between high DPP3 levels and worse respiratory function was also observed, as indicated by worse Horowitz index, namely worse ratio of partial pressure of oxygen in the arterial blood (PaO_2_) to the fraction of inspiratory oxygen concentration (FiO_2_). Furthermore, patients, who experienced deterioration in their total SOFA score and SOFA subscores (liver and renal) during the first 2 days of their ICU stay, presented with high DPP3 plasma concentrations upon admission. Baseline DPP3 levels could also predict organ dysfunction, given that patients with high DPP3 levels on admission were more likely to require invasive mechanical ventilation, renal replacement therapy, as well as more intense and prolonged circulatory support with vasopressors during the first week of hospitalization. When applying serial assessments of DPP3 levels, it was noted that patients who exhibited high DPP3 levels at 24 h were more likely to experience acute kidney injury and deterioration of their total SOFA score within 48 h. Interestingly, this risk was high, irrespective of their baseline DPP3 levels on admission. Indeed, high DPP3 levels at 24 h conferred an increased risk of incident organ dysfunction even in those patients who initially presented with low DPP3 levels upon admission and developed high DPP3 levels at 24 h. Taken together, the authors concluded that DPP3 levels could predict multiple organ failure and reflect patient severity [58].

Besides, similar findings had been previously recorded by Rehfeld et al., who developed two highly specific novel assays for the quantification of DPP3 concentration in human blood samples. To this end, DPP3 plasma concentrations were measured in samples from a subcohort of healthy subjects participating in the Malmö Preventive project, as well as in admission samples from critically ill patients with severe sepsis (175 patients) and septic shock (153 patients) who were randomly selected from the AdrenOSS-1 trial. It was found that both groups of patients with severe sepsis and septic shock exhibited significantly higher levels of DPP3 compared to healthy participants. Furthermore, DPP3 levels were significantly higher in patients with septic shock than in patients with severe sepsis. The corresponding AUCs were 0.7235 for severe sepsis and 0.7927 for septic shock (*p* < 0.0001). It was thus concluded that DPP3 levels not only increase in septic patients compared to healthy subjects, but also reflect the severity of sepsis, since they tend to be significantly higher in patients with septic shock compared to patients with severe sepsis [51].

Along the same lines, Deniau et al. assessed DPP3 plasma levels in 665 ICU patients with various types of shock (septic, cardiogenic, hemorrhagic), the majority of whom (64%—422 patients) were admitted to the ICU due to septic shock. They reported that baseline DPP3 levels were significantly higher in patients with shock than in those without (20.1 [13.6–32.1) ng/mL vs. 17.5 [12.5–26.4] ng/mL). More importantly, they found that high DPP3 plasma levels were associated with the occurrence of organ failure in patients with shock. Indeed, baseline DPP3 levels were higher in patients with shock who developed acute kidney injury (22.7 [14.6–19.6] ng/mL) than in those who did not (16.5 [11.7–23.2] ng/mL). Similarly, DPP3 levels at baseline were significantly higher in patients who required renal replacement therapy (23.2 [14.7–41.2] ng/mL) compared to those who did not (18.7 [12.7–27.4] ng/mL) [62].

In another single-center prospective study from the Netherlands, which included a mixed cohort of ICU patients (11.3% with sepsis), DPP3 levels measured on the first days of ICU admission (days 1, 2 and 3) were found to be associated with all-stage acute kidney injury. This association remained significant even after adjustment for baseline disease severity in multivariate models. It was further noted that patients with more severe stages of acute kidney injury had significantly higher DPP3 concentrations on days 2 and 3. The strongest association with acute kidney injury was observed for DPP3 levels on day 2. Of note, DPP3 levels on days 2 and 3 provided additional predictive value for acute kidney injury to both SOFA and APACHE II scores, whereas DPP3 levels on day 1 added further predictive value only to APACHE II score. In the same study, DPP3 levels, especially on day 2, were found to be correlated with peak levels of other biomarkers of cellular injury, such as aspartate transaminase (AST), alanine transaminase (ALT) and lactate dehydrogonase (LDH). Considering that AST, ALT and LDH reached their peak values at least two days later than DPP3, the latter finding could imply that DPP3 may serve as an early marker of cellular damage during the initial phases of critical illness, including sepsis [63].

DPP3 levels and their association with outcomes was also the subject of investigation in a Swedish trial which included a mixed population of 1978 ICU patients, 32% (632 patients) of whom were admitted due to sepsis. Median DPP3 values for septic patients were 19 [13–31] ng/mL. Elevated DPP3 levels were found to be correlated with all types of organ dysfunction in the whole cohort, as evidenced by higher values of neurological, hepatic, coagulation, renal and cardiovascular SOFA subscores calculated on day 2 after ICU admission. With regard to patients with sepsis, DPP3 levels on admission were significantly associated with the hepatic and coagulation SOFA subscores on day 2, and to a lesser extent with renal and neurological dysfunction. Therefore, elevated DPP3 levels on admission could predict subsequent organ dysfunction in septic patients. The prognostic value of DPP3 was primarily evident for the hepatic and coagulation dysfunction [64].

### 3.3. DPP3 as a Tool for Guiding Treatment

In the AdrenOSS-1 study, it was shown that serial measurements of DPP3 levels in ICU patients during the early phase of sepsis could predict organ dysfunction. Persistently elevated levels of DPP3 were associated with acute kidney injury, worse total SOFA score at 48 h and worse outcomes, whereas early decline of DPP3 levels from high admission values towards normal values could predict improvement of organ function after 1 week. Based on these findings, the authors highlighted the prognostic value of serial DPP3 measurements and concluded that dynamic changes in DPP3 levels could serve as a useful tool for guiding the early management of septic patients in the ICU and enhancing clinical decision-making [58]. Likewise, Deniau et al. suggested that DPP3 levels could help optimize the management of patients with septic shock, based on their findings that high DPP3 levels on admission were associated with organ failure, and specifically with acute kidney injury and the need for renal replacement therapy [62].

Besides, in the study by van Lier et al., when measuring DPP3 levels on three consecutive days (day 1, 2 and 3) after ICU admission in a mixed cohort of critically ill patients, the highest DPP3 concentrations were recorded on day 1 (56.2 [31.8–93.1] ng/mL), whereas DPP3 concentrations on days 2 and 3 were significantly lower (25.7 [16.9–49.7] ng/mL and 30.1 [18.3–67.2] ng/mL, respectively). Moreover, DPP3 levels on the first three days of ICU admission were found to be more strongly associated with acute kidney injury than with mortality. In addition, the authors presumed that DPP3 levels on day 1 primarily reflect disease severity, since the association of baseline DPP3 levels with mortality was no longer evident in models corrected for baseline disease severity. On the contrary, this was not the case for the association of DPP3 levels on day 1 with acute kidney injury, which remained significant in multivariate models even after adjusting for disease severity. When combining these findings with the fact that DPP3 levels are strongly correlated with peak concentrations of cellular injury markers (ALT, AST, LDH), which peak much later than DPP3, it is plausible that DPP3 may serve as a very early marker of cellular damage and organ failure and may thus help clinicians guide management with proper and prompt adjustments of ongoing treatment as early as the septic patient is admitted to the ICU [63].

### 3.4. DPP3 as a Prognosticator of Mortality

In the AdrenOSS-1 study, DPP3 levels were significantly associated with 28-day mortality in ICU patients with sepsis or septic shock. DPP3 levels on admission remained independently associated with 28-day mortality even after adjustment for age, gender, comorbidities, sepsis severity and lactate. More importantly, DPP3 levels on admission had incremental prognostic value over and above APACHE II score, SOFA score, lactate levels or procalcitonin levels. Notably, DPP3 levels on admission were superior to lactate or procalcitonin levels, in terms of predicting short-term mortality. Compared to low DPP3 levels, high baseline DPP3 levels upon admission (>40.4 ng/mL) were associated with significantly worse mortality both at 28 and at 90 days. Patients with high DPP3 levels exhibited a threefold greater risk of death at 28 days. Moreover, during Kaplan–Meier survival analysis based on quartiles of baseline DPP3 concentrations, it was observed that the survival rate of patients presenting with high baseline DPP3 levels above the third quartile (>40.4 ng/mL) dramatically decreased within the first week of ICU hospitalization, showing a much steeper decline compared to the survival curves of patients belonging to lower quartiles. It was also shown that, when baseline DPP3 levels on admission were combined with a second DPP3 measurement at 24 h, further prognostic value for predicting 28-day mortality was added, independently and on top of the admission value. Indeed, patients with decreasing DPP3 values from high baseline levels to low levels at 24 h had a reduced 28-day mortality risk that was comparable to the mortality risk of patients in whom DPP3 levels remained low both at baseline and at 24 h. Conversely, patients whose DPP3 concentrations increased from low baseline levels to high levels at 24 h exhibited an increased 28-day mortality risk, while patients with persistently elevated DPP3 levels at both time points had the worst survival rate. The prognostic value of serial DPP3 measurements remained significant even after adjusting for ICU risk scores (SOFA and APACHE II) and lactate [58]. These results from the AdrenOSS-1 study corroborated previous findings from Rehfeld et al. who reported that in patients with septic shock higher DPP3 levels were accompanied by a higher mortality risk, since DPP3 concentrations were significantly more elevated in non-survivors compared to survivors from septic shock [51].

In a similar fashion, high plasma DPP3 levels were found to be associated with mortality in a mixed cohort of ICU patients with shock (septic, cardiogenic, hemorrhagic). Indeed, results from an analysis of the international multicenter FROG-ICU (French and European Outcome Registry in Intensive Care Units) study pointed towards the fact that high DPP3 levels were strongly correlated with both short- and long-term mortality. Specifically, baseline DPP3 levels were significantly higher in non-survivors (27 [15.7–56.5] ng/mL) compared to survivors (18.5 [12.9–26.7]) ng/mL). During Cox regression analysis, DPP3 levels at baseline were strongly associated with 28-day mortality with a HR of 1.6 [1.5–1.8] for the overall cohort. Regarding the cohort of patients with septic shock, it was observed that patients with baseline DPP3 levels >38.39 ng/mL had an increased risk of dying at 28 days with a HR of 3.3 [2.2–4.7]. Baseline DPP3 levels could predict 28-day mortality with a C-index of 0.64 [0.59–0.69] for the overall cohort and a C-index of 0.63 [0.57–0.69] for the septic shock cohort. Remarkably, it was shown that baseline DPP3 levels could not only predict 28-day mortality but could also provide further predictive value when added to other well established prognostic parameters, such as Simplified Acute Physiology Score (SAPS), SOFA score, lactate or NT-proBNP. Apart from the association between DPP3 levels on admission and 28-day mortality, it was additionally reported that high DPP3 levels upon ICU discharge were predictive of 1-year mortality. Indeed, ICU survivors who were discharged from the ICU with high DPP3 levels were found to be at high risk of dying within the following year with a HR of 3 [1.7–5.4] [62].

In a prospective observational Dutch study, which included a mixed cohort of 650 ICU patients (11.3% of whom had sepsis), DPP3 levels were assessed upon admission (day 1), as well as on days 2 and 3. On univariate Cox regression analysis, it was found that DPP3 levels were significantly associated with 28-day mortality, but only on days 1 and 2 and not on day 3. On multivariate analysis, after adjusting for baseline clinical severity scores (SOFA or APACHE II scores), only DPP3 levels on day 2 were recognized as independent predictors of mortality. In addition to that, DPP3 levels on day 2 were found to add further predictive value with regard to 28-day mortality on top of both SOFA and APACHE II scores [63].

In a multicenter observational study from Sweden, blood samples were prospectively collected upon ICU admission from a mixed cohort of patients admitted to the ICUs of 4 hospitals due to various etiologies, such as sepsis, trauma and cardiac arrest. The aim of the study was to examine the prognostic role of DPP3 levels upon admission with regard to 30-day mortality, which was the primary endpoint. Admission levels of DPP3 were found to be independently associated with 30-day mortality with an OR of 1.30 [1.08–1.57] for the subgroup of septic patients, after correction for SAPS-3 score. In unadjusted models, admission levels of DPP3 alone turned out to be a moderate predictor of 30-day mortality for septic patients, yielding an AUC of 0.62 [0.57–0.67]. When DPP3 was added to SAPS-3 in the logistic regression model, the prognostic capability of the model improved with an AUC of 0.74 [0.70–0.78] for septic patients. The authors concluded that DPP3 is an independent predictor of 30-day mortality and seems to provide additional prognostic information for ICU patients, not captured by SAPS-3 alone [64].

Table 3 summarizes the main characteristics and findings of the studies which have explored the role of DPP3 as a biomarker of risk stratification, prediction of severity, morbidity and mortality, and treatment guidance in patients with sepsis.

## 4. Discussion

Since sepsis remains a major life-threatening condition and a leading cause of in-hospital mortality worldwide [65,66], its prompt diagnosis and management at an early stage upon ED or ICU admission is of critical importance [67,68]. Accordingly, a considerable part of current scientific research focuses on the identification of appropriate biomarkers and clinical scoring systems that would facilitate early diagnosis, risk stratification and effective management of sepsis in a timely manner [6,69,70]. Among several candidate biomarkers of sepsis that have emerged in recent years, this review provides existing evidence on the potential clinical utility of two novel biomarkers, adrenomedullin in its bio-active form (bio-ADM) and dipeptidyl peptidase 3 (DPP3).

**Figure 1:**
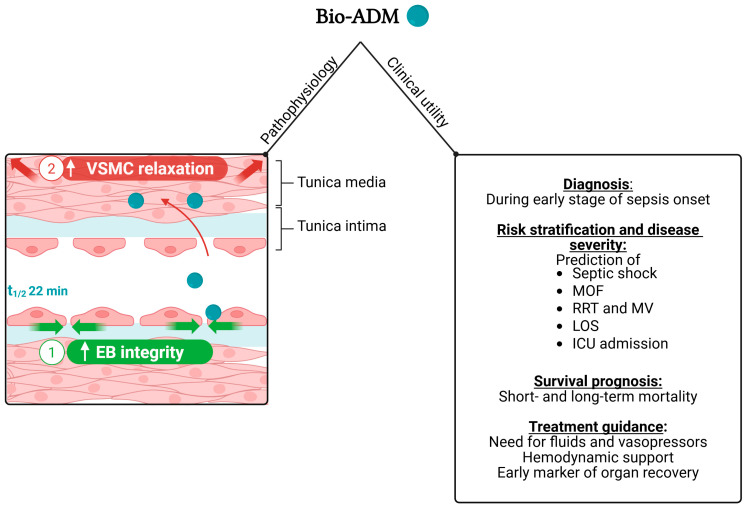
**The role of bio-ADM in vascular homeostasis and its clinical utility in patients presenting to the ED or the ICU with sepsis.** After being released in circulation, bio-ADM displays diverse effects on the vasculature. Bio-ADM binds to its receptors located on the vascular endothelium and results in decreased vascular permeability by promoting the stability of the EB (green arrows), thereby limiting fluid shift towards the extravascular compartment. In parallel, however, bio-ADM moves into the interstitium where it induces relaxation through binding to its receptors located on the VSMCs (red arrows), thereby causing vasodilation and deterioration of systemic hemodynamics [5,10,11,19]. As a biomarker, bio-ADM has been shown to be potentially useful as a diagnostic tool for sepsis [41,42], as a marker of sepsis severity, morbidity and early risk stratification [12,20,26,42,43,44,45,46], as a prognosticator of mortality [12,20,26,41,42,43,44,45,46], and as a tool for treatment monitoring and guidance [26,43]. Abbreviations: Bio-ADM = bioactive adrenomedullin; EB = endothelial barrier; ED = emergency department; ICU = intensive care unit; t_1/2_ = half-life; LOS = length of stay; MOF = multiple organ failure; MV = mechanical ventilation; RRT = renal replacement therapy; VSMC: vascular smooth muscle cell.

When considering the overall management of sepsis, the most promising of these two biomarkers seems to be bio-ADM, since several studies have confirmed its clinical utility as a marker of sepsis severity, morbidity and early risk stratification [12,20,26,42,43,44,45,46], as well as a prognosticator of mortality [12,20,26,41,42,43,44,45,46]. Bio-ADM has also been shown to be potentially useful as a tool for treatment guidance [26,43] and as a diagnostic tool for sepsis [41,42]. Its diagnostic accuracy for sepsis has been reported to range from modest [42] to good [41], when measured at the early stage of sepsis onset. Therefore, it may serve as an early marker for identifying sepsis. Regarding its utility in risk classification and morbidity, bio-ADM has been shown to predict the development of septic shock [43], cardiovascular dysfunction [12,26,42,43] and multiple organ failure, including respiratory, coagulation, hepatic and renal failure [12,20,43]. It has also been associated with a higher incidence of renal replacement therapy [26,42,43], an increased need for mechanical ventilation [45] and an increased length of hospital stay [26,43,45]. Furthermore, it has been correlated with markers of disease severity, such as higher SOFA score [26,43,44], APACHE II score [46] or lactate levels [43,44], and can be used for early stratification of unselected patients presenting to the ED with sepsis by discriminating those who require ICU admission from those who could be discharged from the ED [20]. Bio-ADM has also been demonstrated to predict the need for fluids and vasopressors in terms of both intensity and duration of hemodynamic support [26,43,46]. Accordingly, it may be used as a tool to guide therapy and monitor the efficacy of applied treatment [26,43]. Besides, bio-ADM has been reported to be an early and strong prognosticator of organ recovery and survival, considering that the decrease in bio-ADM concentrations during serial measurements occurs much earlier than the decrease in total SOFA score [26]. Besides, several studies conducted in the ED and ICU setting point towards a prognostic role of bio-ADM in terms of predicting both short-term (28-day or 30-day) [12,20,26,41,42,44,45,46] and long-term (90-day) [43] mortality in patients with sepsis. Of note, some studies have shown that bio-ADM adds incremental prognostic value beyond and on top of established mortality prediction scores [26,42,46], while others have highlighted the prognostic value of repeated bio-ADM measurements during hospitalization [26,41,43,46]. The overall clinical utility of bio-ADM in patients presenting to the ED or the ICU with sepsis is summarized in Figure 1, along with a parallel schematic illustration of the role of bio-ADM in vascular tone and endothelial integrity.

**Figure 2:**
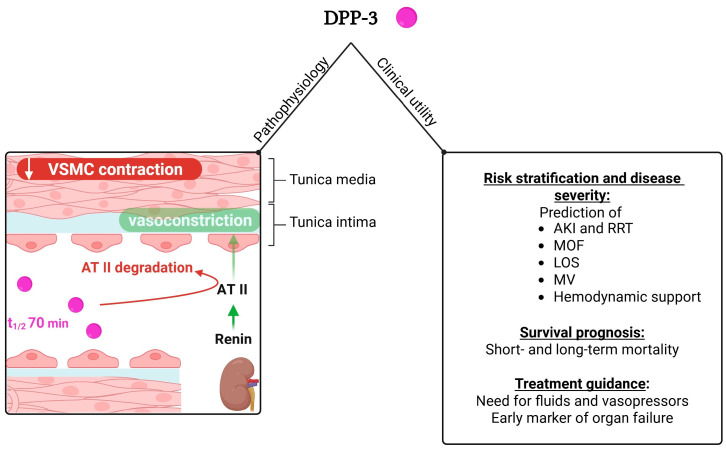
**The role of DPP3 in hemodynamics and its clinical utility in patients presenting to the ED or the ICU with sepsis.** Presumably, after cellular lysis, the cytosolic enzyme DDP3 is released into the circulation, whereby it antagonizes the renin–angiotensin–aldosterone system by cleaving Ang II. As a result of enhanced Ang II degradation by DPP3, all events occurring downstream of Ang II synthesis are inhibited, thereby blunting vasoconstriction of VSMCs and inducing vasodilation. By doing so, DPP3 exerts a negative impact on the hemodynamics of patients with sepsis and/or septic shock [5,48,56,61]. Current literature supports the role of DDP3 in risk stratification of septic patients, in the prediction of sepsis morbidity and mortality [51,58,62,63,64], and in treatment guidance of sepsis and/or septic shock [58,62,63]. Abbreviations: AKI = acute kidney injury; Ang II = angiotensin II; DPP3 = dipeptidyl peptidase 3; ED = emergency department; ICU = intensive care unit; t_1/2_ = half-life; LOS = length of stay; MOF = multiple organ failure; MV = mechanical ventilation; RRT = renal replacement therapy; VSMC: vascular smooth muscle cell.

On the other hand, fewer studies have examined the role of DPP3 in sepsis, all of which have been conducted in the ICU setting. It should be noted that only one trial, the AdrenOSS-1 study [58], included exclusively patients with sepsis or septic shock, whereas all the other trials studied a mixed ICU cohort, including patients with sepsis or septic shock. No study investigated the potential diagnostic role of DPP3 in sepsis. Nonetheless, DPP3 appears to be useful in the risk stratification of septic patients and prediction of morbidity [51,58,62,63,64], survival prognosis [51,58,62,63,64] and treatment guidance [58,62,63]. DPP3 has been shown to predict primarily the development of acute kidney injury and the need for renal replacement therapy [58,62,63]. Regarding the prediction of other organ dysfunctions, DPP3 has also been associated with worse respiratory function and the need for mechanical ventilation [58], circulatory failure and the need for vasopressors [58], as well as hepatic and coagulation dysfunction [64]. In addition, it has been correlated with disease severity, as evidenced by its association with worse metabolic parameters, higher severity prediction scores and prolonged hospital stay [58]. Remarkably, DPP3 has been demonstrated to be an early marker of cellular injury, since its levels reach peak values much earlier than other common markers of cellular damage [63]. Accordingly, DPP3 could serve as a tool for guiding treatment, especially when taking into consideration that serial DPP3 measurements have been shown to predict organ recovery or organ failure depending on the direction of its temporal changes [58]. Besides, the association of high DPP3 levels with positive fluid balance and intensive hemodynamic support with vasopressors further supports DPP3’s potential role in treatment monitoring and guidance [58]. Moreover, it has been demonstrated that DPP3 can predict short-term (28- or 30-day) [58,62,63,64], intermediate-term (90-day) [58] and long-term (1-year) [62] mortality, independently and on top of traditional mortality risk indices [58,62,63,64]. Notably, repeated measurements of DPP3 seem to add further prognostic value in terms of predicting mortality [58]. Figure 2 depicts the clinical utility of DPP3 in patients presenting to the ED or the ICU with sepsis, along with an illustration of its role in hemodynamics.

Nevertheless, certain limitations need to be acknowledged with regard to the studies examining the clinical utility of bio-ADM and DPP3 in patients with sepsis or septic shock. First, patient sample sizes differed among studies. Some studies had small sample sizes [41,46,63], others were moderate-sized [12,44,51], while others used large cohorts of patients [20,26,42,43,45,58,62,64]. The design of the studies was also different, with most of them being prospective [12,20,26,41,43,44,45,46,58,62,63,64] and only one being retrospective [42]. Some trials were single-center studies or registries [12,20,41,44,46,63], whereas others were multicenter [42,43,45,64] or even multinational [26,58,62]. Most studies enrolled exclusively patients with sepsis or septic shock [12,20,26,43,44,46,58], while others included patients from a general ICU population and divided them into sepsis and non-sepsis categories [41,42,62,63,64]. One study recruited patients with clinical signs of suspected life-threatening infection based on a qSOFA score ≥1 [45], while another trial used a group of critically ill ICU patients with sepsis or septic shock and a second group of healthy individuals as a control group [51]. The observational nature of the studies precluded the deduction of cause-and-effect relationships, allowing only descriptions of observed associations between studied biomarkers and outcomes to be made. Another confounding factor was that the recruitment of patients in the studies was based on varying definitions of sepsis and septic shock. The updated Sepsis-3 definitions [2,67] were used in several studies [12,41,42,44,45,62,64], whereas other studies [20,43,46,51,58] were based on former definitions of sepsis and septic shock [71,72,73,74]. One study [26] used mixed definitions, namely the updated ones for sepsis [2] and the former ones for severe sepsis and septic shock [72]. Besides, studies examining the clinical utility of bio-ADM were conducted in different clinical settings. Several studies included exclusively ICU patients [26,41,42,43], other trials were conducted in the ED setting [20,44,45,46], and one study recruited patients from both the ICU and the ED [12]. In contrast, all studies examining the clinical utility of DPP3 were conducted in the ICU setting [51,58,62,63,64], which precludes the generalization of the results to the ED setting. Other than that, the studies differed in terms of primary and secondary endpoints, since each study was designed to investigate diverse outcomes, such as the diagnostic performance of bio-ADM in identifying sepsis [41,42], the association of bio-ADM and DPP3 with mortality [12,20,26,41,42,43,44,45,46,58,62,63,64], with organ failure [12,20,26,42,43,44,58,63,64], with hemodynamic support requirements [26,43,44,58], with the need for renal replacement therapy [26,44,58,62,63] or mechanical ventilation [44,45,58]. Another limitation may be related to the fact that certain studies enrolled patients during daytime or during alternate shifts, which may have led to a selection bias [20,45]. Furthermore, some studies examined dynamic changes in the levels of biomarkers by performing serial biomarker measurements and investigated their impact on outcomes [26,41,43,46,58,62,63], whereas others measured the levels of biomarkers at a single time point (upon admission) and investigated the association of admission levels with outcomes [12,20,42,44,45,64]. Even when studying admission samples only, the non-standardized timing of blood sample collection might have contributed to diverse results, since blood draw was performed within one hour from admission in some studies [20], whereas in other studies blood was drawn within 6 h [64], 12 h [45] or 24 h [12] from the presentation or even the first morning after admission [43]; thus, biomarker levels might have been affected by early study treatment. Moreover, discrepancies among studies could be attributed to the fact that studies used different assays for the measurement of each biomarker [41,44,51,75]. Finally, the variety of cut-offs used for bio-ADM and DPP3 makes the translation of their results to clinical practice really challenging.

It is evident that DPP3 and bio-ADM represent two different biological pathways along the course of sepsis which contribute to the development of septic shock and subsequent organ dysfunction. It is therefore plausible that the combination of both biomarkers would increase the prognostic performance, since each biomarker would provide additional prognostic information not captured by the other one. This hypothesis has been supported by results from the AdrenOSS-1 study showing that the combination of bio-ADM with DPP3 resulted in significant improvement of the C-index for 28-day mortality to a value of 0.742, whereas the corresponding values for each separate biomarker were 0.692 for DPP3 and 0.688 for bio-ADM. It was observed that patients with low levels of both biomarkers (bio-ADM <70 pg/mL and DPP3 <40 ng/mL) exhibited the best 28-day survival rate (92%), whilst patients with elevated concentrations of both biomarkers had the worst 28-day survival rate (54%). In the group of patients with elevated bio-ADM levels but low DPP3 levels, the 28-day survival rate was 78%, whereas those patients with elevated DPP3 levels and low bio-ADM levels demonstrated a survival rate of 71% [5]. Given the multifaceted nature of sepsis and the heterogeneous response to sepsis, this dual biomarker approach could enhance the precision of risk stratification in septic patients, allowing clinicians to tailor interventions based on individual responses [76].

Besides, the evolving landscape of sepsis management necessitates a shift towards personalized medicine. As we move away from a “one-size-fits-all” approach, the identification of patient-specific biomarkers may revolutionize our approach to sepsis management by providing a more comprehensive and sophisticated understanding of the disease [77].

Finally, it is essential to consider the implementation challenges associated with biomarker utilization in clinical practice. While the potential of bio-ADM and DPP3 is promising, barriers such as cost, accessibility and the need for standardization of testing protocols need to be addressed. The development of rapid point-of-care tests that provide reliable results in real-time is crucial for the successful integration of these biomarkers into routine clinical workflows. Additionally, ongoing education and training for the ED and ICU staff on the interpretation and application of these biomarkers will be vital to ensure that they are used effectively in managing septic patients. Collaborative efforts between researchers, clinicians and policymakers will be necessary to create an environment that supports the implementation of these innovative diagnostic tools in everyday clinical practice [78].

## 5. Conclusions

The unacceptably high morbidity and mortality rates of sepsis mandate the utilization of precise and readily available tools that would effectuate early diagnosis, accurate risk stratification and prompt initiation of personalized treatment in septic patients across diverse healthcare settings. To this end, clinicians use a wide combination of clinical parameters, clinical scores and biomarkers that reflect diverse sepsis pathways. Bio-ADM and DPP3 have shown promising results as novel hypoperfusion biomarkers in patients presenting to the ED or the ICU with sepsis or septic shock. Taking into consideration that these biomarkers are implicated in different pathophysiological mechanisms of sepsis, it is not surprising that bio-ADM has been found to be useful for overall sepsis management, from diagnosis and risk stratification to prognosis and treatment guidance, whilst DPP3 has been shown to be mostly valuable in predicting short-term mortality and sepsis-related renal failure. It is plausible that an approach that incorporates the use of these two novel biomarkers, in combination with established risk scores, could lead to the application of refined integrated protocols in the ED and the ICU, which could promptly and effectively inform clinical decision-making in patients presenting with sepsis or septic shock. Given that the translation of current study results into clinical practice remains really challenging, there is no doubt that further high-quality research with large-scale and well-designed randomized clinical trials is warranted in order to reach more robust conclusions on the diagnostic, risk-stratifying and prognostic role of bio-ADM and DPP3 in sepsis.

## Figures and Tables

**Table 1:** Main parameters for the diagnosis of sepsis based on bio-ADM levels.

Study (Author, Year)	Median [IQR] Bio-ADM Concentration			Diagnostic Cut-Off	Sensitivity (%)/Specificity (%)	AUC for Sepsis Diagnosis
	Non-Sepsis	Sepsis	Septic Shock			
Lundberg et al. 2020 [42]	29 pg/mL [18–56]	74 pg/mL [42–145]	107 pg/mL [58–188]	37 pg/mL	61%/80%	0.76 [0.73–0.78]
Yonaha et al. 2021 [41]	2.2 pmol/L [1.2–3.7]	7.8 pmol/L [3.7–18.1]		5.2 pmol/L (≈31.2 pg/mL)	88.1%/67.9%	0.848 [0.773–0.923]

Abbreviations: AUC = area under the curve; bio-ADM = biologically active form of adrenomedullin; IQR = interquartile range.

**Table 2:** Main characteristics and findings of studies exploring the role of bio-ADM in sepsis with regard to diagnosis, risk stratification, prediction of severity, morbidity and mortality, and treatment guidance.

Study (Author, Year, Country)	Study Type Setting	Study Population Demographics	Main Findings
Caironi et al., 2017 Italy [43]	Multicenter open-label randomized trial, ICU setting	Overall cohort: 956 pts; M 58.7% (561) Septic shock cohort: 539 pts (56.4%)	Bio-ADM levels were higher in pts with septic shock. Higher bio-ADM levels at baseline could predict the intensity of hemodynamic support therapy over the first week of treatment, thus serving as a potential tool for guiding hemodynamic treatment. Higher bio-ADM levels were predictive of incident cardiovascular dysfunction, multiple organ failure and shock. Bio-ADM baseline levels and their trajectory over the first week were strongly and independently associated with 90-day mortality.
Mebazaa et al., 2018 Multinational (5 European countries: France, Belgium, Netherlands, Italy, Germany) [26]	Prospective observational multicenter multinational study, ICU setting	Overall cohort: 583 pts; M 62.4% (364); median age 66 [55–76] Sepsis cohort: 290 pts Septic shock cohort: 293 pts	Elevated bio-ADM levels upon ICU admission were strongly correlated with 28-day mortality. Elevated bio-ADM levels were associated with the extent of organ dysfunction (as assessed by the initial SOFA score), as well as with the need for organ support and the duration of organ support. Patients with high bio-ADM levels were more likely to require treatment with vasopressors/inotropes either upon admission or over the following 7 days. Higher bio-ADM levels were also associated with a more positive total fluid balance, higher incidence of RRT and higher ICU length of stay. Early decrease of bio-ADM levels towards normal values within 48 h was associated with recovery of organ function and better 28-day survival.
Lundberg et al., 2020 Sweden [42]	Retrospective observational multicenter study, ICU setting	Overall cohort: 1867 pts; M 60.5% (1129); median age 67 [54–75] Sepsis cohort: 632 pts; M 60.3% (381); median age 69 [61–76]; 267 pts (42.2%) with septic shock Non-sepsis cohort: 1235 pts; M 60.6% (748); median age 65 [49.5–73] Sepsis survivors: 458 pts (72.5%); M 58.5% (268); median age 68 [59–75] Sepsis non-survivors: 174 pts (27.5%); M 64.9% (113); median age 73 [66–79]	Elevated bio-ADM levels on admission were associated with (1) increased 30-day mortality across all cohorts, (2) increased need for dialysis, and (3) higher risk of circulatory failure requiring use of vasopressors. Bio-ADM had incremental prognostic value for 30-day mortality on top of lactate levels among septic pts. After adjustment for disease severity, increased bio-ADM levels were strongly associated with increased risk of having sepsis. The diagnostic accuracy of bio-ADM for identifying sepsis among ICU patients was modest (AUC 0.76). In the overall cohort, a bio-ADM cut-off 70 pg/mL could separate survivors from non-survivors with a specificity of 73% and an NPV of 82% for 30-day mortality. The corresponding specificity and NPV for a cut-off 45 pg/mL were 58% and 84%, respectively. In the sepsis cohort, a bio-ADM cut-off 70 pg/mL had 50% specificity and 77% NPV for 30-day mortality, whereas the corresponding values for the 108 pg/mL cut-off were 68% specificity and 77% NPV.
Lundberg et al., 2022 Sweden [20]	Prospective observational single-center cohort study, ED setting	Overall cohort: 594 pts; M 51.4% (305); median age 73 [61–82] Sepsis survivors: 543 pts; M 50.8% (276); median age 72 [59–82] Sepsis non-survivors: 51 pts; M 56.9% (29), median age 80 [73–88]	High levels of bio-ADM were strongly associated with 28-day mortality. Bio-ADM conferred incremental prognostic value when added to a baseline mortality prediction model. Bio-ADM levels were predictive of ICU admission and development of multiple organ failure. There was an inverse correlation between increasing bio-ADM levels and ED discharge. Low bio-ADM levels were associated with less severe disease and discharge from the ED.
Casalboni et al., 2022 Italy [44]	Prospective observational single-center study, ED setting	Overall cohort: 177 pts; M 44.6% (79), mean age 73.1 ± 17.3 Sepsis survivors: 134 pts; M 55% (74); median age 74 [59–83] Sepsis non-survivors: 43 pts; M 56% (24); median age 85 [79–90]	Bio-ADM levels were significantly associated with 30-day mortality. Regarding short-term mortality, bio-ADM exhibited better prognostic performance than SOFA, NEWS and PENK, while it performed equally to REMS. Bio-ADM levels were significantly correlated with markers of hemodynamic compromise and renal impairment.
Bolanaki et al., 2024 Germany [45]	Prospective observational multicenter study, ED setting	Overall cohort: 1426 pts, M 56% (799); median age 72 [61–80] Sepsis cohort: 417 pts; M 58% (242); median age 74 [63–80]; 14.6% with septic shock Non-sepsis cohort: 1009 pts; M 55.2% (557); median age 71 [59–80]	Bio-ADM provided prognostic information in terms of predicting the need for ICU admission, the need for mechanical ventilation, the length of hospital or ICU stay and 28-day mortality.
Marino et al., 2014 Italy [46]	Prospective observational single-center study, ED setting	Overall cohort: 101 pts; M 60.4% (61); median age 78 [72–83] Sepsis cohort: 72 pts Severe sepsis/septic shock cohort: 29 pts Non-survivors: 29 pts	Baseline bio-ADM levels were associated with disease severity and were negatively correlated with mean arterial pressure. Patients who required vasopressors on admission had significantly higher bio-ADM concentrations than those who did not. Baseline bio-ADM levels could predict 28-day mortality independently of APACHE II, adding further prognostic information on top of APACHE II. Baseline bio-ADM levels were higher in patients dying due to septic shock than in those dying due to other causes. Serial measurements of bio-ADM improved the prognostic performance of bio-ADM.
Kim et al., 2019 Korea [12]	Prospective observational single-center registry, Both ICU and ED setting	Overall cohort: 215 pts; M 59.1% (127); median age 71 [58–79]; 92 ICU pts and 123 ED pts Sepsis cohort: 109 pts; M 59.6% (65); median age 70 [58–79]; 22 ICU pts and 87 ED pts Septic shock cohort: 106 pts; M 58.5% (62); median age 72 [59–79]; 70 ICU pts and 36 ED pts	Bio-ADM levels were significantly associated with septic shock, need of circulatory support with vasopressors, and 30-day mortality rate. There was a strong correlation between bio-ADM quartiles and the number of failing organ systems. Bio-ADM concentrations were significantly associated with cardiovascular, renal, coagulation and liver SOFA subscores. Unlike SOFA cardiovascular subscore and lactate quartiles, only bio-ADM quartiles could consistently predict 30-day mortality and risk-stratify patients both in the overall cohort, as well as within each patient group (sepsis—septic shock).
Yonaha et al., 2021 Japan [41]	Prospective observational single-center study, ICU setting	Overall cohort: 98 Sepsis cohort: 56 pts; M 66% (37); median age 69 [58.5–78] Non-sepsis cohort: 42 pts; M 76% (32); median age 66.5 [56–76]	Bio-ADM levels on admission had the highest diagnostic accuracy for sepsis (in terms of AUC) compared to other sepsis biomarkers (PCT, PSEP, lactate, SOFA score) Bio-ADM was able to predict 28-day mortality on day 3 post-admission, but not at the early onset of sepsis (on day 1). A good correlation exists between levels of total ADM and bio-ADM; hence, both forms may be used interchangeably as reliable early biomarkers for diagnosing sepsis.

Abbreviations: APACHE II = acute physiology and chronic health evaluation II; AUC = area under the curve; bio-ADM = biologically active form of adrenomedullin; ED = emergency department; h = hours; ICU = intensive care unit; M = males; NEWS = national early warning score; NPV = negative predictive value; PCT = procalcitonin; PENK = proenkephalin; PSEP = presepsin; pts = patients; REMS = rapid emergency medicine score; RRT = renal replacement therapy; SOFA = sequential organ failure assessment.

**Table 3:** Main characteristics and findings of studies exploring the role of DPP3 in sepsis with regard to risk stratification, prediction of severity, morbidity and mortality, and treatment guidance.

Study (Author, Year, Country)	Study Type Setting	Study Population Demographics	Main Findings
Blet et al., 2021 Multinational (5 European countries: France, Belgium, Netherlands, Italy, Germany) [58]	Prospective observational multicenter multinational study, ICU setting	Overall cohort: 581 pts; M 62.5% (363); median age 66 [55–75]; 292 pts (50.3%) with septic shock Patients with low DPP3 (<40.4 ng/mL): 436 pts; M 63.5% (277); median age 66 [56–76]; 201 (46.1%) with septic shock Patients with high DPP3 (>40.4 ng/mL): 145 pts; M 59.3% (86); median age 65 [53–75]; 91 pts (62.8%) with septic shock	DPP3 levels upon admission were significantly higher in pts with septic shock (29.1 [18–48.2] ng/mL) than in pts with sepsis (23.2 [15.2–35.1] ng/mL). Baseline DPP3 levels were independently associated with 28-day mortality and added incremental prognostic value on top of APACHE II, SOFA, lactate or PCT. Baseline DPP3 levels could predict short-term mortality better than lactate or PCT. High DPP3 levels at baseline were associated with worse 28- and 90-day mortality. High DPP3 levels at baseline were associated with disease severity (higher SOFA and APACHE II scores), longer ICU stay, higher lactate levels, lower arterial pH, higher NT-proBNP levels, worse renal function and worse respiratory function. Baseline DPP3 levels could predict the need for organ support (vasopressors, fluid volume, MV, RRT). High DPP3 levels on admission could predict a worsening of SOFA score in the first 48 h of ICU stay. Serial DPP3 measurements could predict organ dysfunction and 28-day mortality, thus serving as a potential tool for guiding the early treatment of sepsis.
Rehfeld et al., 2019 Germany [51]	Characterization and validation of 2 novel assays for measuring DPP3 concentration and activity in human plasma	Control group: 2256 healthy participants Sepsis cohort: 175 ICU pts Septic shock cohort: 153 ICU pts; 116 survivors and 37 non-survivors at 28 days	In the healthy population-based cohort, the normal reference range for DPP3 in plasma was 58.6 ± 20.5 U/L. Both sepsis and septic shock cohorts had significantly higher DPP3 levels than the control group. Pts with septic shock demonstrated significantly higher DPP3 levels compared to pts with sepsis. Within the septic shock cohort, DPP3 levels were significantly higher in non-survivors than in survivors.
Deniau et al., 2022 Multinational (France, Germany, Netherlands) [62]	Prospective observational multinational study, ICU setting	Enrollment cohort: 2087 pts Overall cohort of patients with shock: 665 pts; 422 pts (64%) with septic shock; 136 pts (20%) with cardiogenic shock; 107 pts (16%) with hemorrhagic shock Non-survivors from shock at 28 days: 178 pts (47%); 118 with septic shock Non-survivors from shock at 1 year: 317 pts (47%)	Pts with shock had significantly higher DPP3 levels at baseline than pts without shock. Baseline DPP3 levels were significantly higher in non-survivors than in survivors. Baseline DPP3 levels were strongly associated with 28-day mortality and could predict 28-day mortality with a C-index of 0.64 [0.59–0.69] for the overall cohort and a C-index of 0.63 [0.57–0.69] for the septic shock cohort. Baseline DPP3 levels provided incremental predictive value for 28-day mortality on top of established biomarkers (lactate, NT-proBNP, troponin I) and scores (SAPS, SOFA). In pts with septic shock, elevated DPP3 levels at baseline (>38.39 ng/mL) conferred an increased risk of dying at 28 days with an HR of 3.3 [2.2–4.7]. DPP3 levels at ICU discharge were associated with 1-year mortality. Baseline DPP3 levels were associated with the development of AKI and the need for RRT.
van Lier et al., 2023 Netherlands [63]	Prospective observational single-center study, ICU setting	Overall cohort: 650 critically ill pts; M 64.6% (420), median age 65 [55–72] Sepsis cohort: 73 pts (11.3%)	The highest DPP3 levels were observed upon ICU admission (Day 1) and started declining thereafter (Day 2 and 3). DPP3 levels on Day 1 and on Day 2 were significantly associated with 28-day mortality. In multivariate models, after adjusting for disease severity, only DPP3 levels on Day 2 remained significantly associated with 28-day mortality and provided added predictive value on top of SOFA and APACHE II scores. DPP3 levels on Day 1, 2 and 3 were all associated with the development of AKI both in univariate and multivariate models. DPP3 levels on Day 2 demonstrated the strongest association with AKI. DPP3 levels were strongly associated with other biomarkers of cellular damage, i.e., AST, ALT, LDH.
Frigyesi et al., 2021 Sweden [64]	Prospective observational multicenter study, ICU setting	Overall cohort: 1978 mixed ICU pts; M 61%; median age 66 [54–74] Sepsis cohort: 632 pts (32%); M 60%; median age 69 [61–76] Cardiac arrest cohort: 190 pts (9.6%); M 73%; median age 68 [60–76] Trauma cohort: 157 pts (7.9%); M 77%; median age 55 [33–70]	Baseline DPP3 levels could predict 30-day mortality independently of SAPS-3. For the sepsis cohort, DPP3 alone was a moderate predictor of 30-day mortality (AUC 0.62). When adding DPP3 to SAPS-3, the predictive ability improved (AUC 0.74). DPP3 upon admission was associated with subsequent organ dysfunction. In the sepsis cohort, baseline DPP3 was primarily associated with hepatic and coagulation dysfunction and to a lesser degree with renal and neurological dysfunction.

Abbreviations: AKI = acute kidney injury; ALT = alanine transaminase; APACHE II = acute physiology and chronic health evaluation II; AST = aspartate transaminase; AUC = area under the curve; DPP3 = dipeptidyl peptidase 3; ED = emergency department; h = hours; HR = hazard ratio; ICU = intensive care unit; LDH = lactate dehydrogenase; M = males; MV = mechanical ventilation; NT-proBNP = N-terminal pro B-type natriuretic peptide; PCT = procalcitonin; pts = patients; RRT = renal replacement therapy; SAPS = simplified acute physiology score; SOFA = sequential organ failure assessment.

## Data Availability

No new data was created or analyzed in this study.
